# Not all sensors are created equal: a framework for evaluating human performance measurement technologies

**DOI:** 10.1038/s41746-019-0082-4

**Published:** 2019-02-14

**Authors:** Brian Caulfield, Brenda Reginatto, Patrick Slevin

**Affiliations:** 1 The Insight Centre for Data Analytics, O’Brien Centre for Science, University College Dublin, Dublin 4, Ireland; 2 Applied Research for Connected Health, NexusUCD, Belfield Office Park Block 9/10, Clonskeagh, Dublin 4, Ireland; 3 The Insight Centre for Data Analytics, O’Brien Centre for Science, University College Dublin, Dublin 4, Ireland

**Keywords:** Outcomes research, Technology

## Abstract

Recent years have witnessed an explosion in the number of wearable sensing devices and associated apps that target a wide range of biomedical metrics, from actigraphy to glucose monitoring to lung function. This offers big opportunities for achieving scale in the use of such devices in application contexts such as telehealth, human performance and behaviour research and digitally enabled clinical trials. However, this increased availability and choice of sensors also brings with it a great challenge in optimising the match between the sensor and a specific application context. There is a need for a structured approach to first refining the requirements for a specific application, and then evaluating the available devices against those requirements. In this paper we will outline the main features of such an evaluation framework that has been developed with input from stakeholders in academic, clinical and industry settings.

## Introduction

The market availability of digital devices that measure different aspects of human performance and behaviour has significantly increased in recent years. Human performance and behaviour measurement technology refers to consumer and medical grade health and wellbeing devices across a number of fields such as wearable, digital health and remote monitoring technologies. It is estimated that the number of connected wearable devices worldwide will increase from 325 million in 2016 to 929 million by 2021.^1^ Similarly, the digital health consumer base is growing in tandem, and it is forecasted that by 2021, the number of people availing of remote monitoring programmes will grow to 52 million globally.^2^ Although the increased availability of such devices is leading to greater research and commercial opportunity, it can also create significant confusion, especially for professionals who are attempting to select appropriate technologies that meet the requirements of their specific application, whether it is clinical trial, a research study or a digital health service. To the authors’ knowledge, there are currently no standardised methods to help professionals identify, evaluate and compare the numerous human performance devices available with respect to their specific application requirements. In the absence of such a method, several issues exist for professionals who are undertaking device evaluations.

The first of these issues is the need for a tool that helps professionals identify devices that satisfy their application requirements. In many cases, when technologies are chosen and later evaluated, it is often not the device that emerges as the problem per se. It is that the device was, at the time of selection, not appropriate given the specific needs and requirements of the service provider and/or the user. Therefore, to address such an outcome, the authors would argue that the application requirements should be the driver of the process (i.e., device identification, evaluation and comparison). This creates a fresh emphasis for the professional to understand the nuances of their specific application.

Though there are fuzzy boundaries between them, it is useful to consider three primary application contexts for human performance devices: Wellness/Fitness; Healthcare; and Clinical Trials/Research, each with different use cases, depending on the primary motivation for use (Table 1). Each application will have their own particular set of requirements to consider when deciding upon the type of device to deploy there within.

**Table 1:** Three primary application contexts for human performance devices

Application	Use cases
Wellness/Fitness	Personal health/wellness use cases, where the goal is to use data from the device to help a person to better manage their lifestyle.
	Fitness and performance use cases, where the goal is to provide data than can help to guide a training programme for sporting activity.
Healthcare	Behaviour modification use cases, where the goal is to provide input to a structured treatment programme for management of a healthcare issue, or engage patients in their own care process.
	Clinical decision-making process use cases, where the goal is to provide data that can guide diagnosis, treatment decisions or measure outcomes of care.
Clinical trials/Research	Behaviour modification use cases, where the goal is to provide input to a self-directed intervention that might compliment or enhance the impact of a therapeutic product.
	Endpoints, where the goal is to document the impact of a therapeutic product.

To help demonstrate the diverging requirements that can exist between applications in relation to a specific device, Table 2 compares the potential high-level requirements for deploying a wearable activity tracker as part of an employee wellness programme versus those of a clinical trial endpoint.

**Table 2:** Example of high-level requirements for deploying a wearable tracker in different application contexts

Application context	Wellness/Fitness	Clinical trial
Use case	User: healthy middle-aged adult engaging in employee wellness programme in a large multinational organisation	User: volunteer participant in clinical trial
	Provider: employer/health Insurer	Provider: Clinical Trial Organisation
	Goal: monitor activity, sleep, mood and nutrition during a healthy lifestyle promotion initiative. Primary goal is to provide summary and trend reports back to employees to motivate them during programme.	Goal: monitor participant’s activity and sleep during study to provide for accurate measurement of impact of therapeutic intervention.
Device requirements	• Unobtrusive, with deploy and forget capability • Flexible body location • Measurement accuracy desirable, yet not critical • Battery life: >3 months preferable • Automated synchronisation with user’s own data aggregation & communication device (e.g., smartphone) • Automated upload to server.	• Unobtrusive, with deploy and forget capability • Wrist worn location • Validated measurement accuracy, supported by regulatory filing • Battery life: >1 week • Automated synchronisation with provisioned data aggregator • Automated upload to server.

It is evident from this example that the requirements of an application can be more nuanced and complex than one might imagine. The requirements appear similar in both use cases, yet even at this high level there are some critical differences and even more would be likely to emerge on a detailed analysis of the discrete requirements for each use case. This raises an issue for professionals while attempting to choose an appropriate device due to the difficulty of accounting for the plethora of requirements within an application context. For example, professionals may not be familiar with establishing a set of requirements. Or more importantly, they may have questions about the various criteria that are important to define when choosing a device. To help address questions like these, the authors would argue that a process to thoroughly guide professionals through the definition of their application requirements could decrease the risk of selecting a device that does not fully account for the needs of the service or user and are, therefore, not fit for purpose.

Another issue facing professionals in this space is the lack of a holistic tool for evaluating human performance devices. Several tools are available to help professionals evaluate digital health technologies but these tools are heavily biased towards measuring human factor criteria. A reason for this is that many of these tools have been developed within the discipline of human–computer interaction where the evaluation of user interfaces associated with web applications and mobile technologies are a core focus.^3–9^ Elsewhere, tools have also been developed to evaluate the acceptance, user experience and usability of both the hardware and the software aspects of digital systems, products and services.^10–15^ However, while these tools can be useful, there is a need to also evaluate aspects such as regulatory compliance, technical specifications and capabilities and scientific evidence supporting the use of a given device. These evaluation domains can be particularly relevant in highly regulated applications such as clinical trials. Once more, the availability of a holistic evaluation tool, which takes all such aspects into consideration, could support professionals to determine more effectively whether a device is indeed fit for purpose, according to their specific application requirements.

A final issue that should be highlighted is the lack of a tool to evaluate human performance devices prior to their implementation. As mentioned above, the available evaluation tools are primarily focussed on measuring human factor criteria. Because of their nature, as tools that are focussed on the outcome of a person’s interaction with a product, they are frequently administered post-implementation. Yet, it is not until a post-implementation evaluation is conducted that the devices’ appropriateness to the service provider and the end-user is discovered, at which time the device could emerge as not fit for purpose. Long before this point however, a decision to invest in a device, or several devices, was made. Such scenarios illustrate that an opportunity exists for a tool that can help mitigate the risk of spending resources on devices that are not appropriate, by extending the evaluation process to the pre-purchase phase where discrete devices are identified and evaluated against the application requirements so that the most appropriate device can be selected in a systematic and informed manner.

The aim of this paper is to address the gaps highlighted above by describing a framework for evaluating human performance technologies. The framework guides professionals through the processes of defining application requirements, searching for and selecting candidate devices, and finally, performing a structured evaluation of these devices against application requirements—all with a view to helping them determine if a device is fit for purpose and worthy of field testing based on their specific requirements. Whether these requirements are in the context of a clinical trial, a pilot study, or a digital health service, the outcome should reflect a systematic and rigorous evaluation.

## Results

**Fig. 1:**
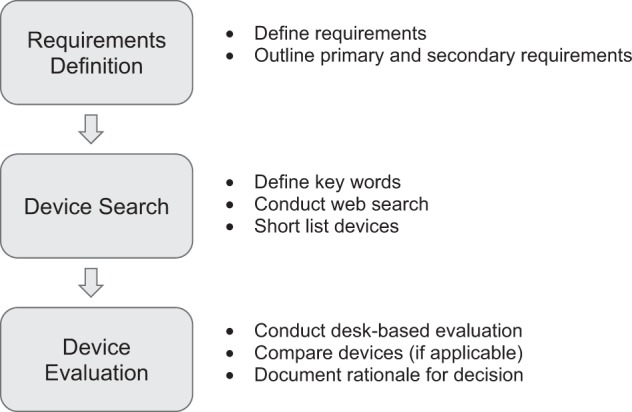
Evaluation framework’s three-step process

The evaluation framework follows a three-step process: (1) Requirements Definition, (2) Device Search and (3) Device Evaluation (Fig. 1). Each step of this framework is supported by relevant templates, which guide the user through the process and to allow for the clear documentation of the rationale for their choice. In this regard, the user is defined as the person/group responsible for selecting the device for deployment in the specified application. Though it is recommended that the framework be employed in a systematic manner, the steps could be applied in sequence or users could elect to apply isolated elements of the framework if constrained by resources and time. For example, there may be situations where one or more devices of interest have already been identified as part of an ad-hoc process. In this case, the user could complete step one (Requirements Definition), skip step two (Device Search) and proceed to step three (Device Evaluation) to determine which of the pre-selected devices is most fit for purpose and/or worthy of field testing, according to their specific requirements. On the other hand, some users may not have the resources or time to enable completion of a formal field testing phase, and therefore this could limit the application of the framework to a desk-based evaluation of identified devices.

### Step 1: Requirements Definition

Defining the application requirements at the beginning of the process enables the user to conduct a more systematic and efficient device search and evaluation. The template provided by the framework guides the user through this process, prompting the consideration of different aspects including: application description and goals, device requirements (e.g., what data need to be collected through the device) and user profile (i.e., who are the people expected to use the device and any specific design requirements they may have). Other aspects to reflect upon include: budget, setting (e.g., home, hospital), geographical location (e.g., urban or rural area), technical requirements (e.g., operational system preferences, compatibility with other equipment and connectivity requirements) and any ethical dilemmas associated with the use of the device (e.g., users are part of a vulnerable population or the device is likely to place undue burden or stress on users). Table 3 offers an excerpt from the requirements definition template.

**Table 3:** Excerpt from the Requirements Definition template

Requirement	Description	Answer
Timeframe	Consider the service/study start date, duration and its conclusion (if applicable). *E.g., Service expected to go live in January 2018. Clients expected to use service during their rehabilitation period (approx. 12 weeks)*.	
Solution description and requirements	Provide a description of the solution required. Consider what you want to achieve with the solution and any specific features or capabilities required. *E.g., activity tracker to monitor compliance with exercise prescription*.	
Data requirements	Provide a description of the data requirements. Consider what data needs to be collected via the solution. *E.g., activity levels (number of steps or distance walked), compliance with exercise programme*.	

Finally, the user is encouraged to categorise requirements as essential or secondary, according to how critical they are to the achievement of the application goals. This is important because it helps users remain grounded in those aspects that are most important, which can be often challenging when evaluating and comparing devices that offer multiple features and functionalities. Additionally, the essential requirements form the basis for the device search strategy, as described below.

It is important to note that the extent and intricacy of the requirements list is at the discretion of the user. A more intricate requirements list will, in general, reduce the pool of devices unearthed in the search, while a high-level requirements list will, in general, broaden the scope of the devices identified.

### Step 2: Device Search

The second step of the framework aims to help the user identify available devices that match their essential application requirements in an efficient and yet comprehensive manner. Firstly, the user is guided through the process of generating keywords based upon the essential application requirements and using such keywords to conduct a systematic web search. Several recommendations are also provided on how to optimise the search, for example, by using particular words or symbols to widen or restrict results, and reviewing the search engine settings to avoid biased results (e.g., based on the user’s location or previous search history).

The user is then prompted to use a comparison matrix template to shortlist devices worthy of a comprehensive desk-based evaluation. It is recommended that only those devices which satisfy all essential requirements are taken to the third step of the framework (Device Evaluation). Table 4 presents an example of the comparison matrix. In this case, only devices 2 and 3 satisfy all essential requirements and are deemed worthy of a comprehensive desk-based evaluation.

**Table 4:** Example of comparison matrix in practice

Essential requirements	Device 1	Device 2	Device 3	Device 4
Wrist worn device	✓	✓	✓	X
Battery life >1 week	✓	✓	✓	✓
iOS and Android compatible	✓	✓	✓	✓
FDA approved	X	✓	✓	X

### Step 3: Device Evaluation

The third step of the framework allows the user to conduct a comprehensive desk-based device evaluation and determine whether one or more devices are worthy of field testing. The template provided prompts the user to answer a number of questions and scrutinise each device according to six domains: (1) Background Information; (2) Cost and Supply Information; (3) Regulatory Compliance; (4) Scientific Evidence; (5) Technical Evaluation; and (6) Human Factors. Table 5 offers an excerpt of the device evaluation template for illustrative purposes. A description of each domain is discussed below.

**Table 5:** Excerpt from the device evaluation template

Technical evaluation									
Component	Query	Device 1 *(insert device name)*	Device 2 *(insert device name)*	Device 3 *(insert device name)*	Device 4 *(insert device name)*	Requirement fulfilled? Y/N/NA/Not clear			
						Device 1	Device 2	Device 3	Device 4
Device specification	(A) What are the device dimensions?								
	(B) What is the device weight?								
	(C) What is the device lifespan?								
Variable measured	(A) What is the target measurement variable?								
	(B) What other variables can be measured?								
	(C) What instruments are in the device (e.g., accelerometer, gyroscope, stress gauge, etc.)?								
Connectivity	(A) E.g. wired, BT, BTLE, Zigbee, Wi-Fi, serial connection?								

#### Background information

The user is prompted to gather background information on the company supplying the device. This may include, for example, information on the size of the organisation, number of years they have been operating, where the company is based and whether they have experienced any product recalls in the past. The goal of this section is to give the user a sense of trust in the company behind the device and clarify whether they possess the required infrastructure to support the use of the device for the purpose specified by the user. Such knowledge can be of critical importance if the application requires a steady supply of a large number of devices.

#### Cost and supply information

This section allows the user to determine whether the device is affordable and available. It covers the costs of the device (including the need for additional or recurrent purchases, shipping fees, or technical support subscription charges), as well as relevant supply information, such as availability in the target country, minimum order requirements and the possibility of obtaining a free sample.

#### Regulatory compliance

This domain requires the user to consider whether the device evaluated complies with relevant regulatory standards, with due regard to the territory or territories in which the device will be deployed. This includes not only safety and performance standards, but also data protection regulation applicable to the target location where the device will be used.

#### Scientific evidence

The user is encouraged to examine the scientific evidence supporting the intended use of the device. This includes, but is not limited to, evidence demonstrating the validity and accuracy of the device’s target measurement in comparison to the gold standard, data quality under field conditions (as opposed to highly controlled, in-lab environments), clinical safety and performance, technical feasibility and usability. Where the intended use stated by the manufacturer differs from the user application, it is important to investigate whether there is evidence supporting the latter. For instance, if the user wishes to use an activity tracker originally designed for athletes with a cohort of geriatric patients, it would be important to determine whether there are any data published on the use of the device by older people.

#### Technical evaluation

This domain scrutinises the device’s technical specifications and capabilities. The intention is to give the user a deep understanding of how the device operates and what technical infrastructure may be required. Examples of sub-sections within this domain include device dimensions, battery life and charging methods, calibration requirements, operational system compatibility, connectivity requirements (e.g., wired, Wi-Fi, BT), data access and storage (e.g., is it possible to access raw data from the device? Where is the data stored?), data security (e.g., how is user data protected?) and data visualisation (e.g., does the device provide feedback? In this case, where is it displayed?).

#### Human factors

The final domain of the evaluation template relates to the device usability and other human factors. The questions in this section help the user examine the level of end-user interaction required, as well as any obvious design issues, which may hinder usability and user experience. Other aspects to consider include the device material (e.g., is it washable? Is it durable? Could it cause allergy or skin irritation?) and the quality of educational materials provided.

#### Data gathering and interpretation

The user may refer to a variety of sources to obtain the information required to complete the device evaluation process. These might include the supplier’s website, news outlets, blogs, scientific journals, discussion forums and communication with the supplier. Where information regarding a query cannot be located, it is recommended that this is clearly stated (e.g., ‘information not found’), instead of making assumptions around the device features and capabilities. Documenting the access date and source of information is also highly encouraged as there may be discrepancies depending on when and where information is garnered (e.g., different resellers may offer different prices). Documenting the information source is also particularly beneficial when revisiting the decision-making process in the future.

Once all queries have been answered, the columns on the right-hand side of the template prompt the user to compare the devices. This can be seen on the ‘Requirements Fulfilled?’ column presented in Table 5. For each query the user should try to determine whether the relevant application requirement is fulfilled for each device. It is recommended to clearly document if the query is not relevant to the user requirements or if further information is required to finalise a decision. Once more, this is beneficial when revisiting the decision-making process in the future.

By comparing how well the devices satisfy the application requirements under each domain, the user should be in a much more informed position to determine which device or devices are worthy of field testing. It is recommended to clearly document the rationale for the decision made, as well as any specific areas that require further investigation through field testing. In the case where a conflict between two devices emerges, and the user is satisfied that they have obtained all the information they can to help inform their desk-based evaluation, it is recommended that the user field tests both devices to determine the most fit-for-purpose device.

## Discussion

The evaluation framework presented in this paper was developed as a collaboration between academic, industry and clinical stakeholders to address the lack of an existing structured approach to help professionals evaluate human performance technologies. The framework provides a comprehensive tool which enables the user to define their specific requirements, conduct a systematic web search and complete a holistic desk-based evaluation, to determine whether one or more devices are fit for purpose and/or worthy of field testing.

The first two steps of the framework, Requirements Definition and Device Search, are unique in comparison to existing resources. To the authors’ knowledge, this is the first tool to prompt users to thoroughly reflect upon and prioritise their requirements prior to selecting a device. It is believed that this will enable users to conduct a more efficient search and grounded evaluation, decreasing the risk of selecting devices that fail to fully account for the specific needs of their application. Similarly, no other resources have been found to support professionals in conducting a systematic web search to identify devices that match such requirements.

While existing tools may help users evaluate specific aspects of a digital health device, these resources are not conducive of a holistic evaluation. The six domains presented on the third step of the framework address this gap by allowing users to conduct a comprehensive desk-based evaluation regardless of their own area of expertise. It is also expected that this exercise may help users identify areas where they require specialist input to help them decide whether a device matches their application requirements.

Finally, it is important to note that the desk-based evaluation process described in this paper is not expected to replace the need for field testing of selected devices. It is, however, believed that it will greatly help users identify critical issues in a timely manner, i.e., before significant time or resources are spent on implementing devices that are not fit for purpose. This offers a significant advantage over existing resources which mainly focus on evaluating devices post-implementation.

## Methods

The three-step evaluation process outlined above was developed using an iterative participatory design approach, as described by Simonsen and Hertzum.^16^ This is a hybrid design approach that emphasises the involvement of potential future end-user’s expertise and experiences primarily for the design of technologies, businesses and social innovative products and services.^16,17^ Moreover, as well as being an inclusive design process, it is also iterative, where researchers and potential future end-users work collaboratively to discover, explain, reflect and integrate knowledge at various time-points in the process to aid in the productive development of the design.^18^

**Fig. 2:**
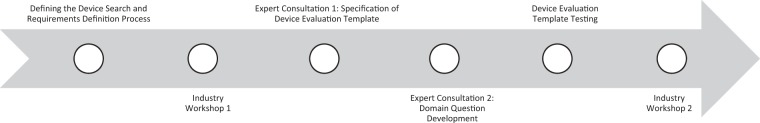
Main project stages and stakeholder input

The approach was felt to be most appropriate considering the cross-collaborative nature of the research which required the input of various types of expertise in the health technology field, from both research and industry, throughout the design process of the evaluation framework. Two industry collaborators were involved at different points throughout the process. In both cases, selection of human performance measurement technologies was a critical issue for their business, one company being involved in telehealth service provision, and the other being a clinical research organisation. Figure 2 illustrates the main project stages including the point at which each stakeholder group was involved.

### Defining the Device Search and Requirements Definition processes

The initial phase of the project aimed to define the Device Search process (step 2 in Results). Early iterations of this process were trialled by the researchers (B.R. and P.S.) using two discrete smart blood pressure monitors as the focus for the desk-based web search. These searches were unstructured but did entail the formulation of keyword searches. When all keyword sequences were saturated, the researchers reconvened to critically evaluate the process used.

The core aspect to emerge from this early work was that although the process made sense in terms of formulating keyword sequences to identify potential devices, without the requirements of a specific use case, the web search findings were too expansive. For instance, a plethora of smart blood pressure monitors were identified, but without contextual information such as a set budget per device, there was no early mechanism to filter down the large number of devices garnered from the web search.

Through further consensus, the researchers decided that hypothetical applications with specific requirements should be developed first, one academic in nature and one from a health technology industry perspective. Doing so would allow the researchers to assess the flexibility of the tool in relation to the diverse needs of potential end-users. Crucially, the specific application requirements would help focus the identification process while providing the device evaluation with a more purposeful direction. A hypothetical academic application, concerning a diabetes self-monitoring study requiring the use of a smart glucometer, was then developed, forming the basis for the Requirements Definition process (step 1 in Results). Ethical approval was obtained from the Ethics research committee at University College Dublin, Ireland. Verbal consent was obtained from participants prior to the commencement of workshops and expert consultations.

### Industry workshop 1

A 1 h workshop with the two industry collaborators was then arranged with the aim of: (a) gaining feedback regarding the overall project design and trajectory and (b) defining an industry-led hypothetical application. During the workshop, the collaborators were presented with the hypothetical academic application use case. Based on this example, they were then given the task of developing an industry-led application use case that reflected the requirements of a provider style study. The primary outcome of the workshop was the development of industry-led hypothetical use case namely a medication adherence programme, requiring the use of a smart pill adherence tracking device.

### Expert consultation 1: specification of device evaluation template

The aim of this phase was to develop the specifications for the device evaluation template. To align with the iterative participatory approach, the research centre’s existing in-house healthcare technology expertise was leveraged to identify the specifications. To provide the basis for the feedback sessions with the expert group (*n* = 7), the authors developed an alpha version of the device evaluation template, using a list of device evaluation criteria that had originally been used by one of them (B.C.) as a teaching tool.

Each expert was invited to participate in a 30-min brainstorming session with researchers (B.R. and P.S.). The experts came from a variety of digital health backgrounds including biomedical, software and systems engineering, human factors, regulatory, clinical and digital health expertise. Each expert was provided with a copy of the alpha version at the beginning of the session. They were asked for their feedback regarding the domain content in the context of using it to evaluate a digital health and wellbeing device. Notes were taken by the researchers regarding the relevant points made by each expert. Upon completing the feedback sessions, the notes gathered were collated and examined for patterns. As patterns were identified, they were cross-checked for consistency and compiled into iteration additions. The researchers then refined the domains and the alpha template was subjected to its first iteration.

### Expert consultation 2: domain question development

The aim of this phase was to develop the questions within each domain. To ensure these iterations complied with initial feedback and comments, the same experts (*n* = 7) were invited to partake in a follow-up 30-min feedback session with two researchers (B.R. and P.S.). The experts received a copy of the iterated template at the beginning of the session and asked for their feedback regarding the domains and questions there within. As the expert critiqued the iterated template, notes were taken, and afterwards collated and examined for patterns. If patterns were identified, they were cross-checked for consistency and compiled into iteration additions. The insights gathered informed the final iteration. The researchers now had a beta version of the template ready for testing.

### Pre-evaluation template testing

In this phase, the researchers (B.R. and P.S.) aimed to define the devices, per hypothetical use case, that would be allocated to the external researchers for testing the device evaluation template. To explain how the devices were chosen for the testing phase, it will be instructive to provide an example using the smart pill-box hypothetical use case. The researchers followed the first two steps of the process: Requirements Definition and Device Search. Leveraging the requirements defined by the industry collaborators (Table 6), the researchers conducted the device web search based on these criteria: smart or connected device; ability to track medication (pill/tablet) adherence; portable device; offline use enabled (i.e., store and forward); potential ability for other services to access; monitoring data (near) real time; currently available for purchase; distributed in Ireland; and compliance with European Union (EU) regulations (CE marking, EU Data Protection Directive).

**Table 6:** Application requirements

Requirement	Description
Application purpose	This medication adherence service will aim at assisting targeted patient populations remain compliant with medication regimens.
	As a first step, the service provider will conduct a pilot study to better understand the impact of smart pill adherence tracking devices on medication compliance levels. The following questions will be addressed:
	1. What are the relevant adherence rates among the 3 study arms: (a) patient uses no device (e.g., conventional plastic bottle/manual adherence tracking); (b) patient uses a pill adherence tracking device without any associated follow-up intervention in the event of non-adherence; and (c) patient uses a pill adherence tracking device with a follow-up intervention in the event of non-adherence. 2. What are the (a) usability and (b) technical/ installation/operational issues associated with the chosen pill adherence tracking device in a home setting?
Cohort profile	Hypertension patients >65 years taking tablets 1x / day.
	Total number of patients across the 3 groups yet to be defined.
Ethnographic analysis	Given that hypertension patients are typically 60 years or older, factors related to ageing would have to be considered (i.e. poorer levels of sight, hearing, and dexterity through the fingers) and will inform decisions regarding device selection.
Setting	Home
Geographical location	Dublin, Ireland
Timeframe	6 Months
Deployment duration	Patients will be asked to track their medication adherence for 2 months.
What data need to be collected (via device)?	Electronic Medication Compliance Data (timestamp).
What type of device is required?	Smart pill adherence tracking device.
What level of accuracy is required?	Medical standard device—device classification (e.g., class I, class II) to be determined as part of the device evaluation process.
Are there any ethical issues to be considered?	None of significance
Standards and regulatory factors to be considered	Compliant with EU standards (CE marking and EU Data Protection Directive).
Hygiene considerations	Devices used require cleaning on return.
Human factors to be considered	Patient: ease of use, reliability, durability.
	Service provision: ease of installation.
	Device: must be portable (e.g., on vacation) and allow offline use.
Technical factors to be considered	Device manufacturer must provide a mechanism for the data to be pulled from their servers (this will allow the service provider to use their own application instead of manufacturer’s app).
	Must allow real-time access to monitoring data by other services.
	Must provide an optional follow-up intervention in the event of non-adherence (group (b): won’t use this feature; group (c): will).
Budget	To be determined as part of the device evaluation process.
In-house expertise available	Design and engineering staff

Using the Google search engine, a search was conducted to identify potentially suitable devices. The researchers went 4 pages deep into the Google search engine (10 results displayed per page) for each keyword permutation. In each case, the first 40 results were examined to search for relevant devices. Once new search combinations were not yielding any new device search information within the 40 results, the researchers concluded that saturation had been reached and ceased the search. The following keywords were used: smart / connected / wireless / Bluetooth; monitor / track; medication / pill / tablet; and adherence / compliance.

A number of keyword permutation combinations were tested. One researcher (B.R.) tested combinations using the keyword ‘monitor’ (e.g., smart+monitor+medication+adherence+device), while another researcher (P.S.) tested combinations containing the word ‘track’ (e.g., smart+track+medication+adherence+device).

The following combinations retrieved the largest number of new results: (smart+monitor+medication+adherence+device); (smart+monitor+pill+adherence+device; wireless+monitor+medication+adherence+device); (Bluetooth+monitor+medication+adherence+device); and (Bluetooth+monitor+medication+compliance+device). A record was not kept of the number of unique devices that were found using each search combination.

From this search, 21 devices were initially identified. All smartphone apps, connected blister packs and smart ingestible pills, totalling 5, were excluded since they were outside the scope of this medication adherence programme. A further two devices were excluded because they were not focussed on pill or tablet adherence monitoring. Another two devices were excluded because they were bound to a service that did not allow integration with external services. The remaining 12 devices were subjected to the requirements comparison matrix. Based on this comparison, 9 devices were excluded because they did not meet one or more criteria or not enough information was available despite contacting manufacturing company and 3 devices were shortlisted as suitable to allocate to the evaluation template testing phase as seen in Table 7.

**Table 7:** Device shortlisting

Core requirements	eCap	GlowCap	Dosecue	iMPack Health Kraken	LineHealth	MedFolio	MEMSCap	Philips Medication Dispensing Service	MedSmart PLUS	AdhereTech	Med-e-lert	Med Signals	SMRxT	uBox	Wisepill
Smart or connected device	Yes	Yes (via AT&T Cellular Network)	Yes	Yes	Yes	Yes	Not clear	Connected to remote monitoring service	Connected to remote monitoring service	Yes	No	Yes	Yes	Yes	Yes
Ability to track medication (pill/tablet) adherence	Yes	Yes	Yes	Yes	Yes	Yes	Yes	Yes	Yes	Yes	No timestamp recorded	Yes	Yes	Yes	Yes
Portable device	Yes	Yes + plugged into power outlet	Yes	Yes	Yes	Yes	Yes	No	Yes, but must be connected to phone line to send data	Yes	Yes	Yes	Yes	Yes	Yes
Offline use enabled (store + forward)	Yes	Not clear	Not clear	Yes	Not clear	No	Yes	No	Yes	Yes	No	Yes	Not clear	Not clear	Yes
Ability of other services to access monitoring data (near) real time	Not clear	Yes	Yes	Not clear	Yes	Yes	Not clear	No	No	Yes	Not clear	Yes	Not clear	Not clear	Yes
Currently available for purchase	Not clear	Yes	Not clear	Not clear	Not clear	Not clear	Not clear	Yes	Yes	Yes	No	Yes	Not clear	No	Yes
Distributed in Ireland	Not clear	Not clear	Not clear	Not clear	Not clear	Not clear	Not clear	No	No	Yes	No	Yes	No	No	Yes
Compliance with EU regulations	Not clear	Not clear	Not clear	Research purpose only	No	Not clear	Not clear	Not clear	No	Yes	Not clear	Yes	No	Not clear	Yes

The same process was followed by the researchers to determine the devices to be allocated to those testing the evaluation template based on the smart glucometer hypothetical use case. In total, 10 devices were identified of which 2 were found to be suitable for the testing phase.

### Evaluation template testing

For this phase, the aim was to finalise the device evaluation template in terms of its usability and general experience. To ensure objectivity, external researchers (*n* = 5) from a range of digital health and wellbeing backgrounds, other than those used in the specification of the device evaluation template, were recruited to test the beta version. Both hypothetical application use cases were tested; two participants were allocated the smart glucometer academic use case, and three were allocated the smart pill-box industry use case. Each participant was emailed a copy of the beta version template, a copy of the application requirements plus an outline of the devices to be evaluated. No parameters were defined for the testing other than to test the device evaluation template using the devices allocated to them. The testing was completed at their convenience.

Upon completion, comments were received via email from the participants in relation to the usability, user experience and perceived usefulness of the evaluation template. Their feedback was collated and examined for patterns. When patterns were identified, they were cross-checked for consistency and compiled into iteration additions. These concluding insights informed the final iteration of the beta version device evaluation template.

### Industry workshop 2

A final 1 h workshop was conducted with the two industry collaborators. The aim was to present the framework and garner final feedback. Particularly, the authors wanted to explore if they felt: (a) that the three-step framework process was a useful and relevant guide and (b) that the device evaluation template was flexible enough to meet their specific needs. Notes were taken and feedback was incorporated to the final version of the evaluation framework.

## Data Availability

Due to the nature of the iterative participatory design approach employed in this study, there is no sharable data-set.

## References

[1] Statista. Connected wearable devices worldwide 2016-2021. https://www.statista.com/statistics/487291/global-connected-wearable-devices/ (2018).

[2] Berg Insights. *mHealth and Home Monitoring* - *8th Edition* (Gothenburg, Sweden: Berg Insight, 2017).

[3] Chin, J. P., Diehl, V. A. & Norman, L. K. Development of an instrument measuring user satisfaction of the human-computer interface. *CHI ‘88 Proceedings of the SIGCHI Conference on Human Factors in Computing Systems* 213–218 (Computer-Human Interaction (CHI) conference, Washington, 1988).

[4] Davis FD (1989). Perceived usefulness, perceived ease of use, and user acceptance of information technology. MIS Q..

[5] Nielsen, J. *Usability Engineering* (Morgan Kaufmann Publishers Inc., San Francisco, 1993).

[6] Lewis JR (1995). Computer system usability questionnaire. Int. J. Hum. Comput. Interact..

[7] Brooke J (1996). SUS - a quick and dirty usability scale. Usability Eval. Ind..

[8] Lin HX, Choong YY, Salvendy G (1997). A proposed index of usability: a method for comparing the relative usability of different software systems. Behav. Inf. Technol..

[9] Madsen, M. & Gregor, S. Measuring Human-Computer Trust. In *Proceedings of the 11th Australasian Conference on Information Systems* (Vol. 53) 6–8 (Australasian Conference on Information Systems, Brisbane, 2000).

[10] Demers L, Weiss-lambrou R, Ska B (2002). The Quebec User Evaluation of Satisfaction with Assistive Technology (QUEST 2. 0): an overview and recent progress. Technol. Disabil..

[11] Mugge R, Schoormans JPL (2006). A longitudinal study of product attachment and its determinants. Adv. Consum. Res..

[12] Tedesco DP, Tullis TS (2006). A comparison of methods for eliciting post-task subjective ratings in usability testing. Usability Prof. Assoc..

[13] Finstad K (2010). Interacting with computers: the usability metric for user experience. Interact. Comput..

[14] Stoyanov SR (2015). Mobile App Rating Scale: a new tool for assessing the quality of health mobile apps. JMIR Mhealth Uhealth.

[15] Hirani SP (2017). Quantifying beliefs regarding telehealth: development of the whole systems demonstrator service user technology acceptability questionnaire. J. Telemed. Telecare.

[16] Simonsen, J. & Hertzum, M. Iterative participatory design (eds J. Simonsen, J. O. Bærenholdt, M. Büscher & J. D. Scheuer) In *Design Research: Synergies from interdisciplinary perspectives*. 16–32 (Routledge, London, 2010).

[17] Manzini E, Rizzo F (2011). Small projects/large changes: participatory design as an open participated process. CoDesign.

[18] Kristensen, M., Kyng, M. & Palen, L. Participatory design in emergency medical service: designing for future practice. In *Proceedings of the 2006 Conference on Human Factors in Computing Systems, Chi 2006* 161–170 (Conference on Human Factors in Computing Systems, CHI, 2006).

